# Next generalization of Cîrtoaje’s inequality

**DOI:** 10.1186/s13660-017-1436-6

**Published:** 2017-07-05

**Authors:** Ladislav Matejíčka

**Affiliations:** Faculty of Industrial Technologies in Púchov, Trenčín University of Alexander Dubček in Trenčín, I. Krasku 491/30, Púchov, 02001 Slovakia

**Keywords:** 26D15, inequalities with power-exponential functions, Cîrtoaje’s inequality

## Abstract

In this paper, we classify sets of solutions of the next generalized Cîrtoaje’s inequality and its reverse, respectively.

## Introduction

In recent years, inequalities with power-exponential functions have been intensively studied [1–7]. They have many important applications. For example, they can be found in mathematical analysis and in other theories like mathematical physics, mathematical biology, ordinary differential equations, probability theory and statistics, chemistry, economics. For more details, a literature review and the history of inequalities with power-exponential functions, see [2]. Cîrtoaje, in [1], has introduced the following interesting conjecture on the inequalities with power-exponential functions. The inequality is similar to the reverse arithmetic-geometric mean inequality where its terms were rearranged.

### Conjecture 1

*If* $a,b \in(0,1]$ *and* $r \in(0,e]$, *then*

1 $$ 2\sqrt{a^{ra}b^{rb}}\geq a^{rb}+b^{ra}. $$

The conjecture was proved by Matejíčka [3]. Matejíčka also proved (1) under other conditions in [4, 5]. For example, he showed that (1) is also valid for $a,b,r \in (0,e]$. In [5], one interesting property of the generalized Cîrtoaje’s inequality (CI) was found. In [6], a classification of sets of solutions of (CI)

2 $$ n\sqrt[n]{\prod_{i=1}^{n}x_{i}^{rx_{i}}} \geq x_{n}^{rx_{1}}+ \sum_{i=1}^{n-1}x_{i}^{rx_{i+1}} $$

was made.

## Methods

In this paper, methods of mathematical and numerical analysis are used. We make a classification of sets of solutions of the other generalization of (CI).

Let *φ*, *ψ* be functions from $\{1,\ldots,n\}$ to $\{1,\ldots,n\}$, where $n\in N$. Put

3 $$ F(r)=\ln n+\frac{r}{n} \Biggl(\sum_{i=1}^{n}x_{\varphi (i)} \ln x_{i} \Biggr)-\ln \Biggl(\sum_{i=1}^{n}e^{rx_{\psi(i)}\ln x_{i}} \Biggr). $$

The function $F(r)$ is defined on $R^{n}_{+}$ where $n\in\mathbf{N}$, $r\geq0$, $R^{n}_{+}=\{(x_{1},\ldots,x_{n}), x_{i}>0, i=1,\ldots,n\}$. We note that $F(r)\geq0$ is equivalent to the following generalization of Cîrtoaje’s inequality (I):

4 $$ n\sqrt[n]{\prod_{i=1}^{n}x_{i}^{rx_{\varphi(i)}}} \geq \sum_{i=1}^{n}x_{i}^{rx_{\psi(i)}}. $$

The reverse inequality to (I)

5 $$ n\sqrt[n]{\prod_{i=1}^{n}x_{i}^{rx_{\varphi(i)}}}< \sum_{i=1}^{n}x_{i}^{rx_{\psi(i)}} $$

we denote by (RI).

## Results and discussion

We remark that in [6] the special case of our classification for (4) was presented, where $\varphi(i)=i$, $\psi(i)=i+1$, $i=1,\ldots,n-1$, $\varphi(n)=n$, $\psi(n)=1$.

The following functions:

6 $$\begin{aligned}& g(x_{1},\ldots,x_{n}) = \frac{1}{n}\sum _{i=1}^{n}x_{\varphi(i)}\log (x_{i})-m_{x}, \end{aligned}$$

7 $$\begin{aligned}& \text{where}\quad m_{x} = \max_{1\leq m\leq n}\bigl\{ x_{\psi(m)}\log (x_{m})\bigr\} , \\& h(x_{1},\ldots,x_{n}) = \sum _{i=1}^{n} (x_{\varphi(i)}-x_{\psi (i)} ) \log(x_{i}), \end{aligned}$$

we will call characteristic functions of (I).

Put

$$ S^{n} =\bigl\{ (x_{1},\ldots,x_{n})\in R^{n}_{+}; x_{i}=x_{j}, i,j=1,\ldots,n \bigr\} . $$

We prove the following lemma.

### Lemma 1

*Let* $F(r)$ *be defined by* (3). *Let* *φ*, *ψ* *be arbitrary functions from* $\{1,\ldots,n\}$ *to* $\{ 1,\ldots,n\}$, $n\in N$. *Then* $F(r)$ *is a concave function for each* $A\in R^{n}_{+}-S^{n}$, *and* $F(0)=0$. *If there is* $i\neq j$; $i,j\in N$ *such that* $x_{\psi(i)}\ln x_{i}\neq x_{\psi(j)}\ln x_{j}$, *then* $F(r)$ *is a strongly concave function in* *A*.

### Proof

$F(0)=0$ is evident. Easy calculation gives

$$ F'(r)=\frac{1}{n} \Biggl(\sum_{i=1}^{n}x_{\varphi(i)} \ln x_{i} \Biggr)-\frac{\sum_{i=1}^{n}e^{rx_{\psi(i)}\ln x_{i}}x_{\psi(i)}\ln x_{i}}{ \sum_{i=1}^{n}e^{rx_{\psi(i)}\ln x_{i}}} $$

and

$$ F''(r)=\frac{-L(r)}{ (\sum_{i=1}^{n}\exp (rx_{\psi(i)}\ln x_{i} ) )^{2}} $$

where

$$\begin{aligned} L(r)&=\sum_{i=1}^{n}\sum _{j=1}^{n}\exp (rx_{\psi(i)}\ln x_{i}+rx_{\psi(j)}\ln x_{j} )x_{\psi(i)}^{2} \ln^{2} x_{i} \\ &\quad {}-\sum_{i=1}^{n}\exp (rx_{\psi(i)}\ln x_{i} )\sum_{j=1}^{n}\exp (rx_{\psi(j)}\ln x_{i} ) (x_{\psi(i)}\ln x_{i})x_{\psi(j)}\ln x_{j} \\ &=\frac{1}{2}\sum_{i=1}^{n}\sum _{j=1}^{n}\exp (rx_{\psi(i)}\ln x_{i}+rx_{\psi(j)}\ln x_{j} )x_{\psi(i)}^{2} \ln^{2} x_{i} \\ &\quad {}+\frac{1}{2}\sum_{i=1}^{n}\sum _{j=1}^{n}\exp (rx_{\psi(i)}\ln x_{i}+rx_{\psi(j)}\ln x_{j} )x_{\psi(j)}^{2} \ln^{2} x_{j} \\ &\quad {}-\sum_{i=1}^{n}\exp (rx_{\psi(i)}\ln x_{i} )\sum_{j=1}^{n}\exp (rx_{\psi(j)}\ln x_{i} ) (x_{\psi(i)}\ln x_{i})x_{\psi(j)}\ln x_{j} \\ &=\sum_{i=1}^{n}\sum _{j=1}^{n}\exp (rx_{\psi(i)}\ln x_{i}+rx_{\psi (j)}\ln x_{j} ) (x_{\psi(i)}\ln x_{i}-x_{\psi(j)}\ln x_{j} )^{2}\geq0. \end{aligned}$$

The proof is completed. □

Now we prove the following lemma.

### Lemma 2

*Let* *g*, *h* *be defined by* (6), (7). *Let* *φ*, *ψ* *be arbitrary functions from* $\{1,\ldots,n\}$ *to* $\{1,\ldots,n\}$, $n\in N$. *Then there are five cases*.

1. *If* $h(x_{1},\ldots,x_{n})=\sum_{i=1}^{n} (x_{\varphi (i)}-x_{\psi(i)} )\log(x_{i})<0$ *for* $A=(x_{1},\ldots,x_{n})\in R^{n}_{+}$ *then* (RI) *is valid for all* $r>0$ *in* $A=(x_{1},\ldots,x_{n})\in R^{n}_{+}$.
2. *If* $h(x_{1},\ldots,x_{n})=\sum_{i=1}^{n} (x_{\varphi (i)}-x_{\psi(i)} )\log(x_{i})=0$ *and* $g(x_{1},\ldots,x_{n})= \frac{1}{n}\sum_{i=1}^{n}x_{\varphi(i)}\log (x_{i})-\max_{1\leq m\leq n}\{x_{\psi(m)}\log(x_{m})\}<0$ *for* $A=(x_{1},\ldots,x_{n})\in R^{n}_{+}$ *then* (RI) *is valid for all* $r>0$ *in* $A=(x_{1},\ldots,x_{n})\in R^{n}_{+}$.
3. *If* $h(x_{1},\ldots,x_{n})=\sum_{i=1}^{n} (x_{\varphi (i)}-x_{\psi(i)} )\log(x_{i})=0$ *and* $g(x_{1},\ldots,x_{n})= \frac{1}{n}\sum_{i=1}^{n}x_{\varphi(i)}\log (x_{i})-\max_{1\leq m\leq n}\{x_{\psi(m)}\log(x_{m})\}=0$ *for* $A=(x_{1},\ldots,x_{n})\in R^{n}_{+}$ *then* $F(r)=0$ *for* $r\geq0$ *in* $A=(x_{1},\ldots,x_{n})\in R^{n}_{+}$.
4. *If* $h(x_{1},\ldots,x_{n})=\sum_{i=1}^{n} (x_{\varphi (i)}-x_{\psi(i)} )\log(x_{i})>0$ *and* $g(x_{1},\ldots,x_{n})= \frac{1}{n}\sum_{i=1}^{n}x_{\varphi(i)}\log (x_{i})-\max_{1\leq m\leq n}\{x_{\psi(m)}\log(x_{m})\}\geq0$ *for* $A=(x_{1},\ldots,x_{n})\in R^{n}_{+}$ *then* (I) *is valid for all* $r\geq0$ *in* $A=(x_{1},\ldots,x_{n})\in R^{n}_{+}$.
5. *If* $h(x_{1},\ldots,x_{n})=\sum_{i=1}^{n} (x_{\varphi (i)}-x_{\psi(i)} )\log(x_{i})>0$ *and* $g(x_{1},\ldots,x_{n})= \frac{1}{n}\sum_{i=1}^{n}x_{\varphi(i)}\log (x_{i})-\max_{1\leq m\leq n}\{x_{\psi(m)}\log(x_{m})\}<0$ *for* $A=(x_{1},\ldots,x_{n})\in R^{n}_{+}$ *then there is* $r_{0}>0$ *such that* (I)*is valid for* $r\in(0,r_{0}]$ *and* (RI) *is valid for* $r\in(r_{0},\infty)$ *in* $A=(x_{1},\ldots,x_{n})\in R^{n}_{+}$.

### Proof

The proof is evident. It follows from Lemma 1. □

### Note 1

It is easy to see that if $g(x_{1},\ldots,x_{n})=0$ and $h(x_{1},\ldots,x_{n})=0$ then $F(r)=0$ for all $r\geq0$. Really, from Lemma 1 we have $F'(0)=0$ and $\lim_{r\rightarrow\infty }F'(r)=0$. If $F(r_{1})\neq0$ for some $r_{1}>0$ then $F(r_{1})<0$. Then there exists *z* such that $F(r_{1})-F(0)=F'(z)r_{1}$ and $0< z< r_{1}$. It implies $F'(z)<0$. Because of $F''(r)\leq0$ we get $F'$ is non-increasing for $r\geq0$. For $r>z>0$ we obtain $F'(r)\leq F'(z)$ so $\lim_{r\rightarrow\infty}F'(r)\leq F'(z)<0$. This is a contradiction.

## Conclusion

In this paper, we showed the following. If (I) is valid in $(x_{1},\ldots,x_{n})$ for some $r_{0}>0$ then (I) is valid in $(x_{1},\ldots,x_{n})$ for all $0< r\leq r_{0}$. Similarly, if (RI) is valid in $(x_{1},\ldots,x_{n})$ for some $r_{0}>0$ then (RI) is valid in $(x_{1},\ldots,x_{n})$ for all $r>r_{0}$.

We think that the way how to classify sets of solutions of the power-exponential inequalities could be used for other suitable inequalities.

Now we give examples of concrete applications of our results. We make the complete classification of sets of solutions for (I) and (RI) inequalities where $n=2$. Using Matlab for plotting graphs of the solution curves for the characteristic equations $g(X)=0$, $h(X)=0$ we obtain the following figures for (I) and (RI). In the figures we denote by $I+\mathit{RI}$ the points where (I) and also (RI) are locally valid. By *I* we denote points where (I) is valid for all $r>0$ and by *RI* we denote points where (RI) is valid for all $r>0$.

It is easy to show that for $n=2$ there are only 12 basic cases of inequalities (I). The other four cases of (I) can be transformed to the previous cases.

### Example 1

Let $n=2$, $\varphi(1)=1$, $\varphi(2)=2$, $\psi(1)=1$, $\psi(2)=2$. Then we have (I):

8 $$ \begin{gathered} 2\sqrt{x_{1}^{rx_{1}}x_{2}^{rx_{2}}} \geq x_{1}^{rx_{1}}+x_{2}^{rx_{2}}, \\ h(x_{1},x_{2})=(1/2) (x_{1}-x_{1} ) \log(x_{1})+(1/2) (x_{2}-x_{2} ) \log(x_{2})=0, \\ g(x_{1},x_{2})=(1/2) \bigl(x_{1} \log(x_{1})+x_{2}\log(x_{2}) \bigr)-\max \bigl\{ x_{1}\log(x_{1}),x_{2}\log(x_{2}) \bigr\} . \end{gathered} $$

See Figure 1.

**Figure 1:**
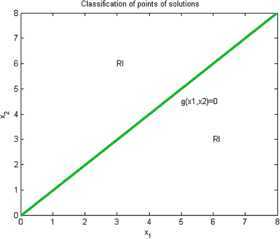
**Solution points for inequalities Examples** **1** **,** **7** **,** **8** **,** **11** **.**

### Example 2

Let us consider that $n=2$, $\varphi(1)=2$, $\varphi(2)=1$, $\psi (1)=1$, $\psi(2)=2$. Then by (I)

9 $$ \begin{gathered} 2\sqrt{x_{1}^{rx_{2}}x_{2}^{rx_{1}}} \geq x_{1}^{rx_{1}}+x_{2}^{rx_{2}}, \\ h(x_{1},x_{2})=\frac{1}{2} (x_{2}-x_{1} )\log(x_{1})+\frac {1}{2} (x_{1}-x_{2} ) \log(x_{2})=\frac{1}{2} (x_{2}-x_{1} )\ln \biggl(\frac{x_{1}}{x_{2}} \biggr), \\ g(x_{1},x_{2})=(1/2) \bigl(x_{2} \log(x_{1})+x_{1}\log(x_{2}) \bigr)-\max \bigl\{ x_{1}\log(x_{1}),x_{2}\log(x_{2}) \bigr\} . \end{gathered} $$

See Figure 2.

**Figure 2:**
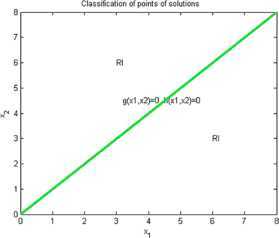
**Solution points for inequalities Example** **2** **.**

### Example 3

Put $n=2$, $\varphi(1)=1$, $\varphi(2)=2$, $\psi(1)=1$, $\psi(2)=1$. Then we obtain by (I)

10 $$ \begin{gathered} 2\sqrt{x_{1}^{rx_{1}}x_{2}^{rx_{2}}} \geq x_{1}^{rx_{1}}+x_{2}^{rx_{1}}, \\ h(x_{1},x_{2})=\frac{1}{2} (x_{1}-x_{1} )\log(x_{1})+\frac {1}{2} (x_{2}-x_{1} ) \log(x_{2}), \\ g(x_{1},x_{2})=(1/2) \bigl(x_{1} \log(x_{1})+x_{2}\log(x_{2}) \bigr)-\max \bigl\{ x_{1}\log(x_{1}),x_{1}\log(x_{2}) \bigr\} . \end{gathered} $$

See Figure 3.

**Figure 3:**
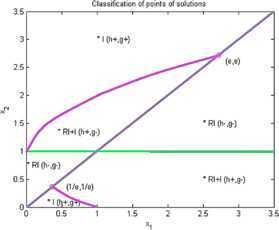
**Solution points for inequalities Example** **3** **.**

### Example 4

Let us consider $n=2$, $\varphi(1)=2$, $\varphi(2)=1$, $\psi(1)=1$, $\psi(2)=1$. Then we get by (I)

11 $$ \begin{gathered} 2\sqrt{x_{1}^{rx_{2}}x_{2}^{rx_{1}}} \geq x_{1}^{rx_{1}}+x_{2}^{rx_{1}}, \\ h(x_{1},x_{2})=(1/2) (x_{2}-x_{1} ) \log(x_{1})+(1/2) (x_{1}-x_{1} ) \log(x_{2}) \\ \hphantom{h(x_{1},x_{2})}=(1/2) (x_{2}-x_{1} )\log(x_{1}), \\ g(x_{1},x_{2})=(1/2) \bigl(x_{2} \log(x_{1})+x_{1}\log(x_{2}) \bigr)-\max \bigl\{ x_{1}\log(x_{1}),x_{1}\log(x_{2}) \bigr\} . \end{gathered} $$

See Figure 4.

**Figure 4:**
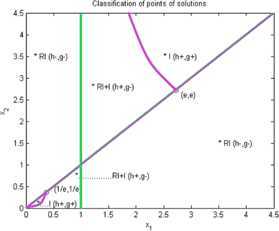
**Solution points for inequalities Example** **4** **.**

### Example 5

Let $n=2$, $\varphi(1)=1$, $\varphi(2)=1$, $\psi(1)=2$, $\psi(2)=2$. Then we have by (I)

12 $$ \begin{gathered} 2\sqrt{x_{1}^{rx_{1}}x_{2}^{rx_{1}}}\geq x_{1}^{rx_{2}}+x_{2}^{rx_{2}}, \\ h(x_{1},x_{2})=(1/2) (x_{1}-x_{2} ) \log(x_{1})+(1/2) (x_{1}-x_{2} ) \log(x_{2}) \\ \hphantom{h(x_{1},x_{2})}=(1/2) (x_{1}-x_{2} )\log(x_{2}x_{1}), \\ g(x_{1},x_{2})=(1/2) \bigl(x_{1} \log(x_{1})+x_{1}\log(x_{2}) \bigr)-\max \bigl\{ x_{2}\log(x_{1}),x_{2}\log(x_{2}) \bigr\} . \end{gathered} $$

See Figure 5.

**Figure 5:**
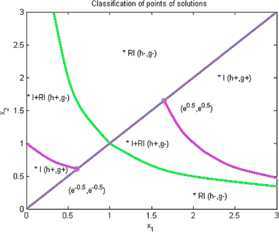
**Solution points for inequalities Example** **5** **.**

### Example 6

Put $n=2$, $\varphi(1)=1$, $\varphi(2)=2$, $\psi(1)=2$, $\psi(2)=1$. Then by (I)

13 $$ \begin{gathered} 2\sqrt{x_{1}^{rx_{1}}x_{2}^{rx_{2}}} \geq x_{1}^{rx_{2}}+x_{2}^{rx_{1}}, \\ h(x_{1},x_{2})=(1/2) (x_{1}-x_{2} ) \log(x_{1})+(1/2) (x_{2}-x_{1} ) \log(x_{2}) \\ \hphantom{h(x_{1},x_{2})}=(1/2) (x_{2}-x_{1} )\log \biggl(\frac{x_{2}}{x_{1}} \biggr), \\ g(x_{1},x_{2})=(1/2) \bigl(x_{1} \log(x_{1})+x_{2}\log(x_{2}) \bigr)-\max \bigl\{ x_{2}\log(x_{1}),x_{1}\log(x_{2}) \bigr\} . \end{gathered} $$

See Figure 6.

**Figure 6:**
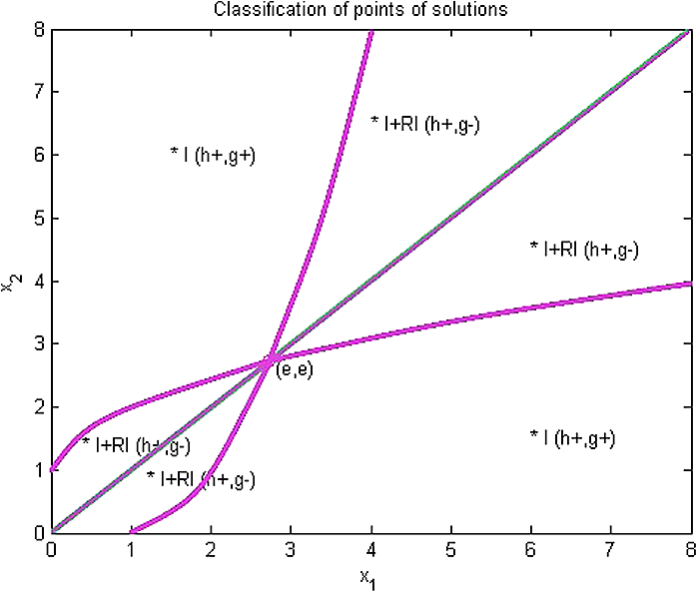
**Solution points for inequalities Example** **6** **.**

### Example 7

Let us consider $n=2$, $\varphi(1)=1$, $\varphi(2)=1$, $\psi(1)=1$, $\psi(2)=1$. Then we obtain by (I)

14 $$ \begin{gathered} 2\sqrt{x_{1}^{rx_{1}}x_{2}^{rx_{1}}}\geq x_{1}^{rx_{1}}+x_{2}^{rx_{1}}, \\ h(x_{1},x_{2})=(1/2) (x_{1}-x_{1} ) \log(x_{1})+(1/2) (x_{2}-x_{2} ) \log(x_{2})=0, \\ g(x_{1},x_{2})=(1/2) \bigl(x_{1} \log(x_{1})+x_{1}\log(x_{2}) \bigr)-\max \bigl\{ x_{1}\log(x_{1}),x_{1}\log(x_{2}) \bigr\} . \end{gathered} $$

See Figure 1.

### Example 8

Put $n=2$, $\varphi(1)=2$, $\varphi(2)=2$, $\psi(1)=2$, $\psi(2)=2$. Then by (I)

15 $$ \begin{gathered} 2\sqrt{x_{1}^{rx_{2}}x_{2}^{rx_{2}}} \geq x_{1}^{rx_{2}}+x_{2}^{rx_{2}}, \\ h(x_{1},x_{2})=(1/2) (x_{1}-x_{1} ) \log(x_{1})+(1/2) (x_{2}-x_{2} ) \log(x_{2})=0, \\ g(x_{1},x_{2})=(1/2) \bigl(x_{2} \log(x_{1})+x_{2}\log(x_{2}) \bigr)-\max \bigl\{ x_{2}\log(x_{1}),x_{2}\log(x_{2}) \bigr\} . \end{gathered} $$

See Figure 1.

### Example 9

Let us consider $n=2$, $\varphi(1)=1$, $\varphi(2)=1$, $\psi(1)=1$, $\psi(2)=2$. Then we get by (I)

16 $$ \begin{gathered} 2\sqrt{x_{1}^{rx_{1}}x_{2}^{rx_{1}}}\geq x_{1}^{rx_{1}}+x_{2}^{rx_{2}}, \\ h(x_{1},x_{2})=\frac{1}{2} (x_{1}-x_{1} )\log(x_{1})+\frac {1}{2} (x_{1}-x_{2} ) \log(x_{2})=\frac{1}{2} (x_{1}-x_{2} )\ln x_{2}, \\ g(x_{1},x_{2})=(1/2) \bigl(x_{1} \log(x_{1})+x_{1}\log(x_{2}) \bigr)-\max \bigl\{ x_{1}\log(x_{1}),x_{2}\log(x_{2}) \bigr\} . \end{gathered} $$

See Figure 7.

**Figure 7:**
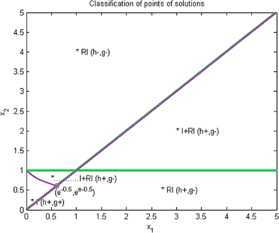
**Solution points for inequalities Example** **9** **.**

### Example 10

Let $n=2$, $\varphi(1)=1$, $\varphi(2)=1$, $\psi(1)=2$, $\psi(2)=1$. Then by (I)

17 $$ \begin{gathered} 2\sqrt{x_{1}^{rx_{1}}x_{2}^{rx_{1}}}\geq x_{1}^{rx_{2}}+x_{2}^{rx_{1}}, \\ h(x_{1},x_{2})=\frac{1}{2} (x_{1}-x_{2} )\log(x_{1})+\frac {1}{2} (x_{2}-x_{2} ) \log(x_{2})=\frac{1}{2} (x_{1}-x_{2} ) \log(x_{1}), \\ g(x_{1},x_{2})=(1/2) \bigl(x_{1} \log(x_{1})+x_{1}\log(x_{2}) \bigr)-\max \bigl\{ x_{2}\log(x_{1}),x_{1}\log(x_{2}) \bigr\} . \end{gathered} $$

See Figure 8.

**Figure 8:**
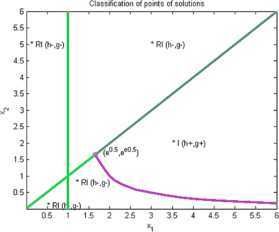
**Solution points for inequalities Example** **10** **.**

### Example 11

Put $n=2$, $\varphi(1)=2$, $\varphi(2)=1$, $\psi(1)=2$, $\psi(2)=1$. We obtain by (I)

18 $$ \begin{gathered} 2\sqrt{x_{1}^{rx_{2}}x_{2}^{rx_{1}}} \geq x_{1}^{rx_{2}}+x_{2}^{rx_{1}}, \\ h(x_{1},x_{2})=(1/2) (x_{1}-x_{1} ) \log(x_{1})+(1/2) (x_{2}-x_{2} ) \log(x_{2})=0, \\ g(x_{1},x_{2})=(1/2) \bigl(x_{2} \log(x_{1})+x_{1}\log(x_{2}) \bigr)-\max \bigl\{ x_{2}\log(x_{1}),x_{1}\log(x_{2}) \bigr\} . \end{gathered} $$

See Figure 1.

### Example 12

Let $n=2$, $\varphi(1)=2$, $\varphi(2)=2$, $\psi(1)=2$, $\psi(2)=1$. Then we have by (I)

19 $$ \begin{gathered} 2\sqrt{x_{1}^{rx_{2}}x_{2}^{rx_{2}}} \geq x_{1}^{rx_{2}}+x_{2}^{rx_{1}}, \\ h(x_{1},x_{2})=(1/2) (x_{2}-x_{2} ) \log(x_{1})+(1/2) (x_{2}-x_{1} ) \log(x_{2})=(1/2) (x_{2}-x_{1} ) \log(x_{2}), \\ g(x_{1},x_{2})=(1/2) \bigl(x_{2} \log(x_{1})+x_{2}\log(x_{2}) \bigr)-\max \bigl\{ x_{2}\log(x_{1}),x_{1}\log(x_{2}) \bigr\} . \end{gathered} $$

See Figure 9.

**Figure 9:**
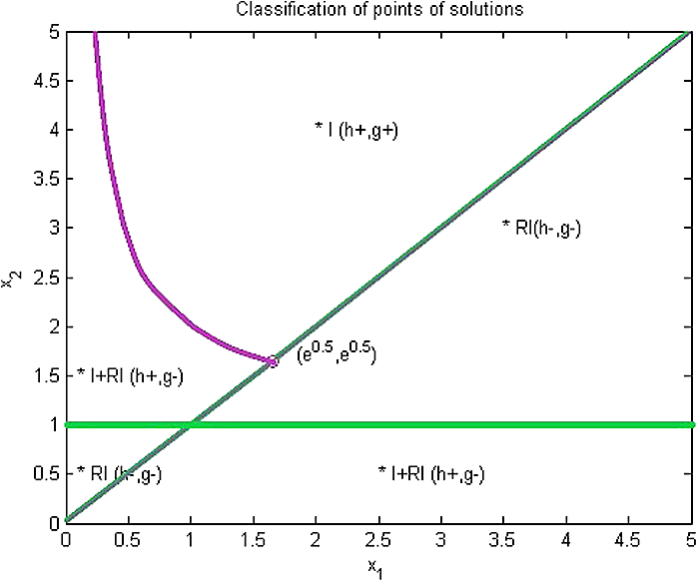
**Solution points for inequalities Example** **12** **.**
